# Reducing Antibiotic Drug Use Effects on *Clostridioides difficile* Colonization in Nursing Home Residents^1^

**DOI:** 10.3201/eid3211.260743

**Published:** 2026-11

**Authors:** Majerle Reeves, Mihnea R. Mangalea, Camden D. Gowler, Prabasaj Paul, Hannah Wolford, Joseph D. Lutgring, Sujan C. Reddy, Alison Laufer Halpin, L. Clifford McDonald, Rachel B. Slayton

**Affiliations:** Centers for Disease Control and Prevention, Atlanta, Georgia, USA (M. Reeves, M.R. Mangalea, C.D. Gowler, P. Paul, H. Wolford, J.D. Lutgring, S.C. Reddy, A.L. Halpin, L.C. McDonald, R.B. Slayton); US Public Health Service, Rockville, Maryland, USA (A.L. Halpin, R.B. Slayton).

**Keywords:** *Clostridioides difficile*, gut microbiota, *Clostridioides difficile* colonization, CDI, modeling, antibiotic drugs, bacteria, bacterial infection, nursing homes, United States

## Abstract

Antibiotic drug–mediated changes in the human gut microbiota affect *Clostridioides difficile* colonization. We created a compartmental model of *C. difficile* colonization in nursing home residents to improve understanding of how the gut microbiota mediates the transmission process. When analyzing *C. difficile* colonization as a function of the daily percentage of the nursing home resident population on antibiotic drugs, the model displayed increases in *C. difficile* colonization as antibiotic drug use increased. Reducing the daily percentage of the nursing home resident population receiving antibiotic drugs from 8% to 6.6% could halve endemic colonization. In sensitivity analyses, increases in the time to recover microbiota diversity, duration of colonization, daily percentage of the nursing home resident population on antibiotic drugs, and susceptibility to *C. difficile* in the low-diversity microbiota population had the largest effect on endemic colonization. Our model demonstrates that reductions in antibiotic drug use yield reductions in *C. difficile* colonization.

*Clostridioides difficile* poses a serious threat to persons within healthcare systems, especially nursing home residents and long-term acute care patients (*1*). Both direct and indirect factors contribute to *C. difficile* colonization risk (Figure 1). Antibiotic drug use can disrupt the gut microbiome and lead to a decrease in microbial diversity and subsequent loss of protection against pathogens (*2*,*3*). Previous studies have demonstrated how antibiotic drug use and duration (*4*,*5*), in addition to gut microbiome status (*3*), affect a person’s risk for colonization and infection by *C. difficile* (*6*).

**Figure 1:**
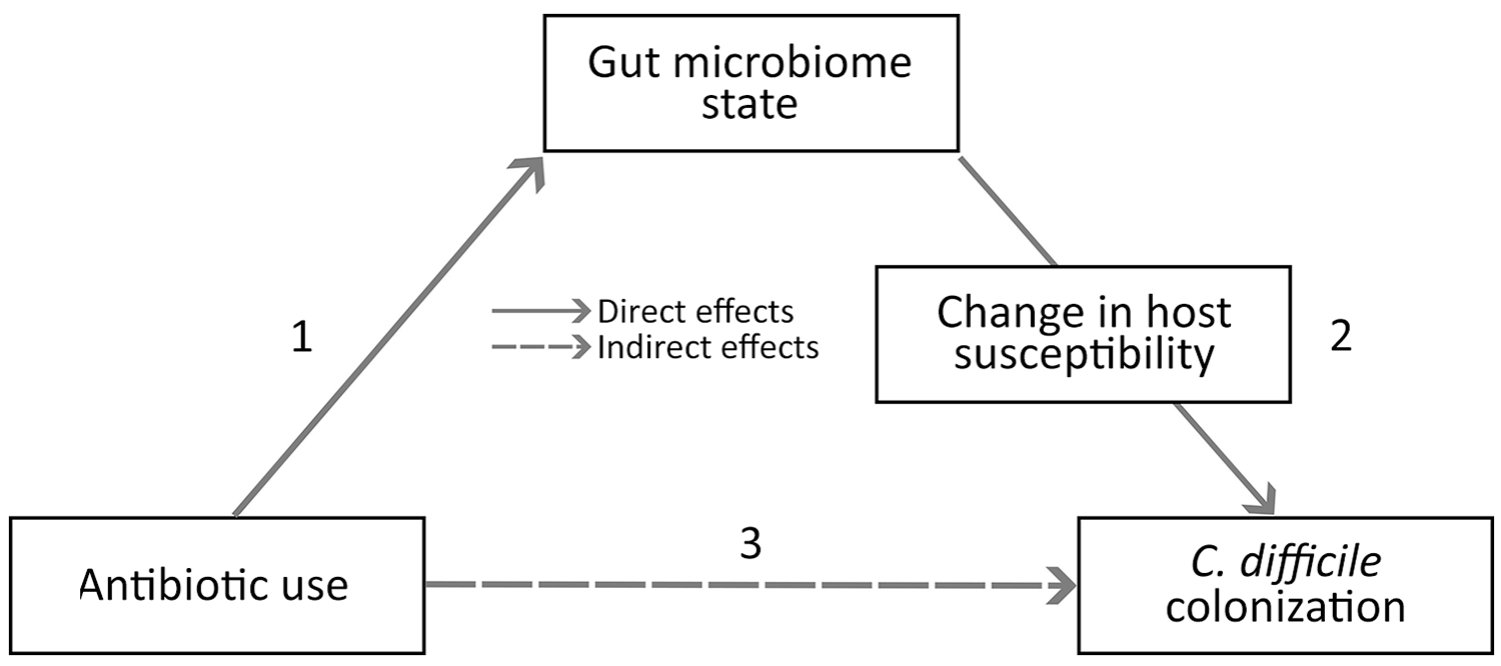
Diagram showing the relationships between antibiotic drug use, gut microbiome state, and *Clostridioides difficile* transmission in study of antibiotic drug use effects on the microbiome state and endemic *C. difficile* colonization and transmission in nursing homes. Increasing antibiotic drug use could lead to higher *C. difficile* transmission through indirect effects, fully mediated by gut microbiota. The negative direct effects of antibiotic drugs on gut microbiota are related to changes in the gut microbiome state that influence host susceptibility to C. *difficile*, leading to an overall indirect effect of antibiotic drugs on C. *difficile* colonization.

One meta-analysis found that antibiotic drug use within 3 months of the study period was not significantly associated with *C. difficile* colonization, although several studies within the analysis found that prior hospitalization, within 6 months of the study period, was associated with increased colonization risk (*7*). Those results suggest 2 possible explanations: collinearity between hospitalization and antibiotic drug use widened the CIs for antibiotic drug use and reduced statistical significance, or antibiotic drug use increased colonization risk only when accompanied by elevated *C. difficile* exposure at the facility level (*7*).

Person-level links between antibiotic drug use and C. *difficile* colonization and infection cannot quantify the population-level consequences of antibiotic drug-induced microbiota disruption and the drivers of transmission within a facility or population (*2*–*5*). Together, those factors highlight the difficulty of disentangling the direct and indirect effects of antibiotic drug use on population level effects versus exposure to *C. difficile* in a person, underscoring the need to explicitly account for microbiome disruption. Consolidating that information to elucidate C. *difficile* transmission and the potential effects of public health interventions remain a challenge that mathematical models are well-suited to address (*8*). We built a model of antibiotic drug use, microbiota dynamics, and *C. difficile* transmission to estimate the magnitude of those indirect effects and to quantify the population-level colonization effect attributable to microbiome-mediated transmission. 

## Methods

### Model Design

We developed a compartmental model (*9*) of *C. difficile* colonization in nursing home residents in which microbiome status affects the transmission process (Figure 2). For microbiome status, we classified the population into high and low ecologic diversity microbiota groups, or α diversity, which quantifies bacterial species’ relative abundance in a microbial community and is a commonly reported metric of microbial community diversity, especially in studies associating colonization (*6*,*10*) or antibiotic drug use (*11*–*14*; P. Ferretti, unpub. data, https://doi.org/10.7554/eLife.90111.1) with the microbiome. The rate of transition from the high to low microbiota diversity group is proportional to the daily percentage of the nursing home resident population on antibiotic drugs. This structure reflects the well-established observation that low microbiota diversity is a risk factor for *C. difficile* colonization (*15*). Within each high and low α diversity group, we further stratified the population as either uncolonized or colonized with *C. difficile*.

**Figure 2:**
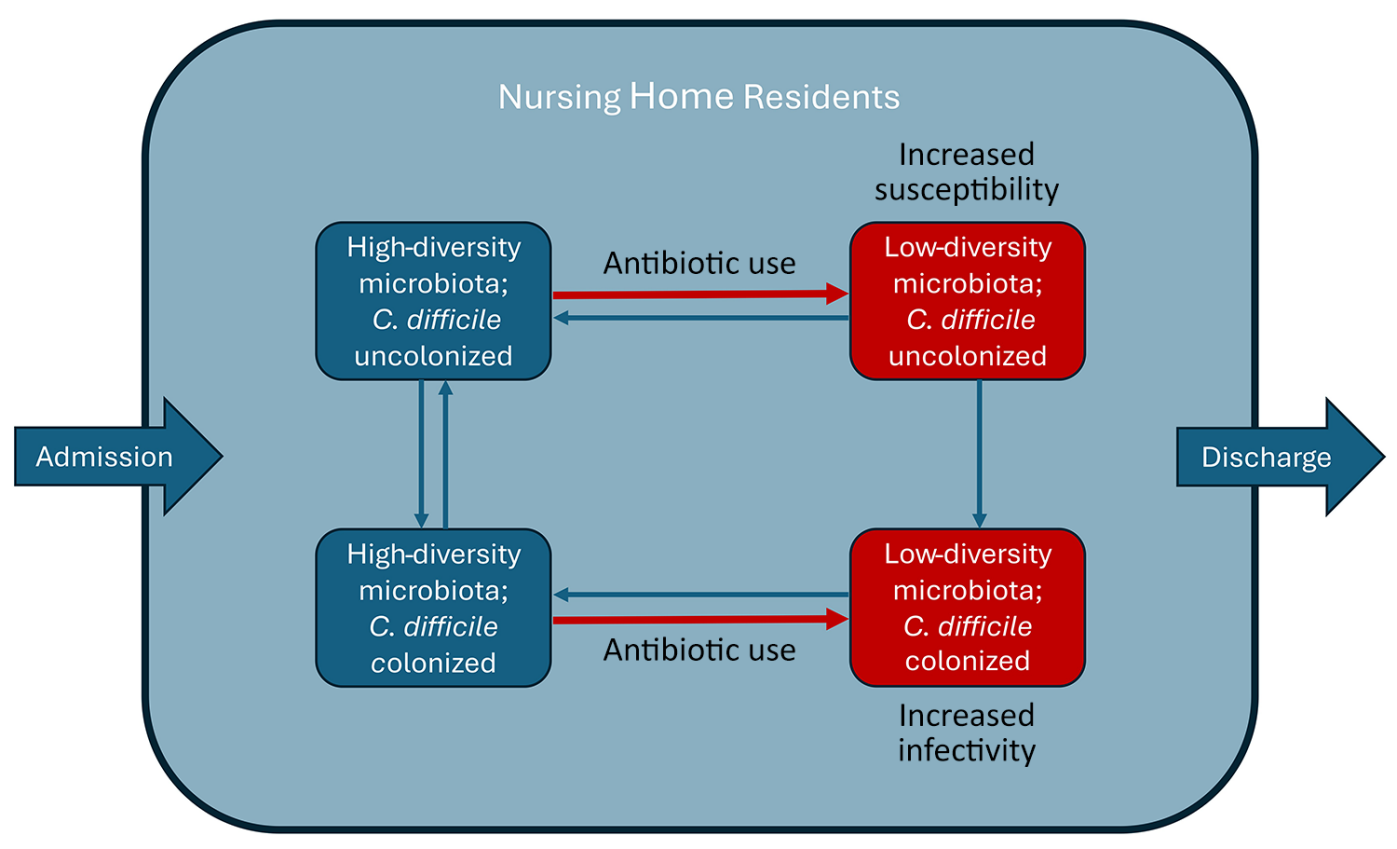
Simplified diagram of a compartmental model in study of indirect effects of antibiotic drug use on the microbiome state and the implications for endemic *Clostridioides difficile* colonization and transmission in nursing homes. Each box represents states nursing home residents can fall under. Each arrow denotes transitions between different states (states of high and low microbiota diversity and uncolonized and colonized with *C. difficile*). The size of the population in the high and low microbiota states is a function of the daily percentage of the nursing home resident population on antibiotic drugs.

Transmission dynamics followed a susceptible-infectious-susceptible framework. The low microbiota diversity groups are more susceptible to *C. difficile* colonization and are more infectious if colonized with *C. difficile*. The *C. difficile*–colonized, low-diversity microbiota group takes more time to recover microbiota diversity than the uncolonized counterparts. The *C. difficile*–colonized, high-diversity microbiome group can spontaneously decolonize, but the *C. difficile*–colonized, low-diversity microbiota group cannot We chose this framework to reflect how well C. *difficile* has evolved to survive in the gut (Table; Appendix, Figure 1).

**Table:** Full parameter table for compartmental model of *Clostridioides difficile* colonization transmission in nursing home residents*

Description	Variable or parameter notation	Modeled value range	Sources
Initial and newly admitted proportion of population with low-diversity microbiota	*b_L_*	0–0.5	(*6*,*10*)
Total population size	*N*	50–200	Assumed
Proportion of *C. difficile*–colonized newly admitted nursing home residents	μ	0–0.02	Assumed
Transmission rate	β	NA	Fitted value
Relative increase in *C. difficile* infectivity because of low diversity microbiota	θ	1–2.5	Assumed
Relative increase in susceptibility to colonization with *C. difficile* for low-diversity microbiota group	ω	1–5	Assumed
Spontaneous *C. difficile* decolonization indicator for low-diversity microbiota group	η	0	Assumed
Antibiotic drug-induced transition rate from high to low diversity microbiota	α†	0.0014–0.07	Estimated
Daily nursing home resident population receiving antibiotic drugs, %	*abx*	2–14	(*16*,*17*)
High to low microbiota diversity after starting antibiotic drugs, d	ψ	2–15	(*13*)
Microbiota diversity recovery rate from low to high for *C. difficile* uncolonized group	δ‡	0.0215–0.065	Estimated
Daily nursing home resident population not on antibiotic drugs, %	1 – *abx*	86–98	(*16*,*17*)
Recovery from low to high microbiota diversity when discontinuing antibiotic drugs, days	*r_d_*	15–40	(*13*)
Duration of *C. difficile* colonization, d	1/γ	24–90	(*15*,*18*)
Multiplier of microbiota diversity recovery for the *C. difficile* colonized group	ϕ	0.75–1	Assumed
Average length of nursing home stay, d	1/ζ	25–406	(*19*)

*NA, not applicable. †α = (daily percentage of the nursing home resident population on antibiotic drugs) × (1/days to go from high to low diversity upon starting antibiotic drugs). ‡δ = (daily percentage of the nursing home resident population not on antibiotic drugs) × (1/days to recover from low to high diversity upon discontinuing antibiotic drugs)

Both increased susceptibility and infectivity increase the colonization rate, which is the force of infection in the model. Although increases in susceptibility and infectivity because of low microbiota diversity are supported by several studies (*6*,*20*,*21*), their magnitude is unknown and therefore assumed. Increased susceptibility increases the colonization rate in the uncolonized, low-diversity microbiota group, reflecting reduced colonization resistance to C. *difficile* when fewer resource-competing microorganisms are present in the gut. Increased infectivity increases the colonization rate in both the uncolonized, high-diversity microbiota group and uncolonized, low-diversity microbiota groups. This effect is akin to the *C. difficile*–colonized, low-diversity microbiota group shedding more *C. difficile* spores, either from a higher *C.* *difficile* bacterial load relative to competing microorganisms or higher transmissibility because of symptomatic infection in a portion of the population. We set a modest increase in infectivity for the *C.* *difficile*–colonized, low-diversity microbiota group well below the 15–30-fold greater infectiousness that previous transmission models attribute to symptomatic infection over asymptomatic colonization (*21*). Because we were modeling colonization dynamics rather than symptomatic *C. difficile* infection, and because susceptibility is mechanistically established, we assume susceptibility has a larger effect on the colonization rate than infectivity in the low-diversity microbiota groups.

### Microbiota-Mediated Indirect Effects

We first investigated microbiota-mediated indirect effects of antibiotic drug use (Figure 1). We used literature-informed parameter ranges to account for and analyze how the microbiota mediates the effect of antibiotic drug use on colonization risk in the nursing home population (Table). In our model, the low α diversity group could have higher susceptibility and infectivity and no spontaneous decolonization. We included parameter values for a comparator model in which the microbiome has no effect on *C. difficile* colonization (Appendix Table 1).

### Model Equilibria Dynamics

Using our model, we analyzed the equilibrium (i.e., endemic) dynamics, and all results reveal the equilibrium colonization prevalence (Appendix). We calculated the endemic colonization prevalence in response to varying the daily percentage of the nursing home resident population on antibiotic drugs. We assumed the baseline daily percentage of the nursing home resident population receiving antibiotic drugs to be 8% (*17*) and the percentage colonized at equilibrium to be 10% (*22*). We varied the daily percentage of the nursing home resident population receiving antibiotic drugs from 2% to 14% and calculated the proportion colonized with C. *difficile* at equilibrium. That range denotes a symmetric 75% decrease and increase in the daily percentage of the nursing home resident population receiving antibiotic drugs; we chose this range as reasonable because total elimination of antibiotic drugs is not feasible. The daily percentage of the nursing home resident population receiving antibiotic drugs affects movement from high to low microbiota diversity and from low to high microbiota diversity. The bidirectional effect of antibiotic drug use creates a nonlinear relationship between antibiotic drug use and the proportion of the population with low-diversity microbiota. Because of the wide ranges for many of the model parameters (Table), we fit 500 scenarios to capture the potential variation in endemic C. *difficile* colonization and report the median, 50%, and 95% simulation intervals for the 500 model scenarios (Appendix). 

### Parameter Effects and Uncertainty

Our compartmental model substantially simplifies *C. difficile* colonization, microbiota diversity, and antibiotic drug use. We chose a simple model because of limited data on pathogen natural history and within host ecologic interactions. We aim to use mathematical modeling to help inform future data collection efforts. With an eye toward applied public health study design, we used the uncertainty in model parameters to evaluate their contribution to model outputs.

We increased individual model parameters and calculated the increase in endemic colonization prevalence and the reduction in transmission rate required to offset the difference between the baseline and new colonization prevalence. This reduction in the transmissibility parameter represents efforts to disrupt transmission, such as hand hygiene with soap and water, contact precautions, or related infection prevention and control interventions. We fit 500 scenarios to capture the potential variation and report the median value and the 50% distribution of estimated values (Appendix).

## Results

### *C. difficile* Comparative Equilibrium Colonization Prevalence

In our model, increasing the daily percentage of the nursing home resident population on antibiotic drugs generated higher endemic colonization prevalence; decreasing the daily percentage of the nursing home resident population on antibiotic drugs reduced endemic colonization prevalence. When looking at endemic colonization prevalence by microbiota diversity status, the proportion of each group colonized as a function of antibiotic drug use increased at a faster rate in the low-diversity microbiota group compared with the high-diversity microbiota group (Figure 3; Appendix Figure 2). The model predicts that a reduction of 17.5% to the daily percentage of the resident population receiving antibiotic drugs, from 8% to 6.6%, would halve median colonization prevalence to 5% (95% CI 2.1%–8.0%). 

**Figure 3:**
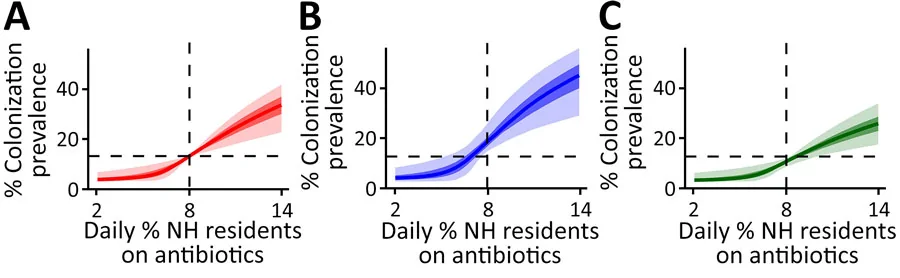
Model results from study of the indirect effects of antibiotic drug use on the microbiome state and the implications for endemic *Clostridioides difficile* colonization and transmission in nursing homes. A) Total population; B) low microbiota diversity group; C) high microbiota diversity group. Dashed horizontal lines represent endemic *C. difficile* colonization at 10% total colonization prevalence when daily population-level antibiotic drug use is 8% (dashed vertical lines). The darker and lighter shaded regions show the central 50% and 95% simulation intervals, and the darkest solid lines depict median values. NH, nursing home.

### Quantifying Parameter Uncertainty

In the sensitivity analysis, we sampled from parameter ranges as defined by the literature (Table). Uniformly sampled parameters (Appendix Figure 3) revealed some weak correlations (Appendix Figure 4). To gauge the effect of each parameter, we individually increased parameters and calculated endemic C. *difficile* colonization. Many parameters had considerable effects on median C. *difficile* colonization prevalence (Figure 4, panel A). For example, increasing the daily percentage of the nursing home resident population receiving antibiotic drugs by 5% in the model led to a median 15.3% increase in colonization prevalence in the simulated nursing home (Figure 4, panel A). Although the uncertainty around the estimated change in colonization prevalence is substantial (interquartile range [IQR] 10.8%–20.0%) (Figure 4, panel A), the change corresponds to a roughly 2–4-fold increase in the percent of residents colonized per percentage increase in the daily percentage of the nursing home resident population receiving antibiotic drugs.

**Figure 4:**
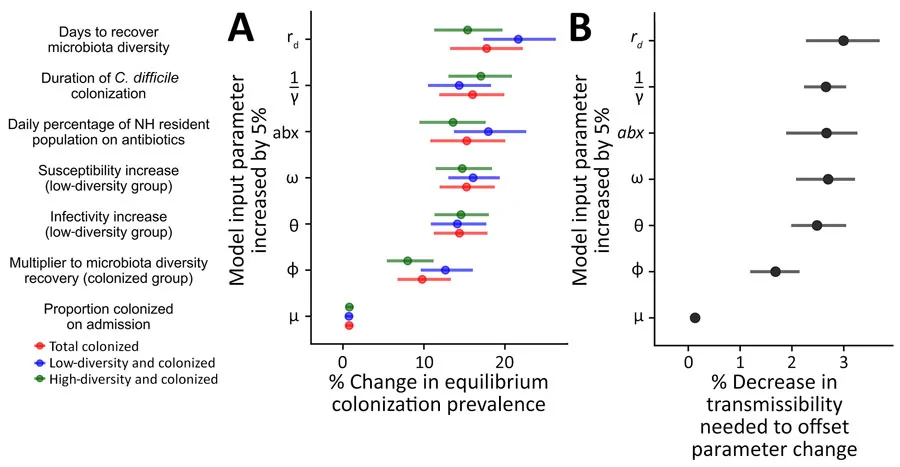
Results from study of the indirect effects of antibiotic drug use on the microbiome state and the implications for endemic *Clostridioides difficile* colonization and transmission in nursing homes. A) Changes in colonization prevalence; B) and the percentage decrease in transmissibility required to return the population to its original *C. difficile* colonization prevalence after a perturbation. Points show the medians, and error bars mark the central 50% distribution of estimated values.

### Microbiome State and Transmissibility

Parameters in the model tying the microbiome state and transmissibility (i.e., relative increases in infectivity or susceptibility because of low-diversity microbiota) had both high effect and wide uncertainty estimates (infectivity, median 14.4%, IQR 11.2%–17.8%; susceptibility, median 15.3%, IQR 11.9%–18.7%) (Figure 4, panel A). Parameters linking *C. difficile* colonization and microbiome state also had both high effect and wide uncertainty estimates. Increasing the microbiota diversity recovery time had the largest effect (median 17.7%, IQR 13.2%–22.2%), followed by the duration of colonization (median 16.0%, IQR 11.9%–19.9%) (Figure 4, panel A). In addition, parameters associated with low-diversity microbiota, such as the daily percentage of the nursing home population receiving antibiotic drugs (median 15.3%, IQR 10.8%–20.0%) and the multiplier to microbiota diversity recovery for the colonized group (median 9.8%, IQR 6.8%–13.3%) had large effects on colonization prevalence with wide uncertainty, largely following the transmissibility parameter set patterns (Figure 4, panel A).

We next quantified the percent reduction in the transmission rate parameter required to restore the original *C. difficile* colonization prevalence after the model input parameters increased by 5%. The same parameters affected in the sensitivity analysis revealed a similar qualitative pattern when individually evaluated (Figure 4, panel B). For instance, increasing antibiotic drug use overall by 5% would require a median 2.7% (IQR 1.9%–3.3%) reduction in transmissibility (e.g., increased contact precautions) to offset the parameter change and return the population to its original, lower colonization prevalence. The rankings and relative magnitude of each parameter were comparable to colonization results (Figure 4, panel A). The central 50% of estimates for most parameters also exhibited considerable uncertainty for the required decrease in transmissibility (Figure 4, panel B).

## Discussion

Our modeling study demonstrates that the host microbiota diversity is critical in *C. difficile* colonization prevalence at the population level. In our model, colonization prevalence increased in response to increased antibiotic drug use, and those nonlinear increases are driven by the nonlinear relationship between antibiotic drug use and the proportion of the population with low-diversity microbiota. That effect is compounded because susceptibility and infectivity are linked to that proportion. Because the microbiome mediates the relationship, low-diversity microbiota represents a modifiable risk factor for *C. difficile* colonization, much like antibiotic drug use (*4*,*23*,*24*), but one that can be directly targeted. Our results therefore highlight the potential for microbiome-targeted therapeutics and pathogen reduction approaches to restore or strengthen *C. difficile* colonization resistance (*25*). Mechanistically, such interventions could decrease the nonlinearity between antibiotic drug use and the proportion of the population with low-diversity microbiota or reduce the effect of low-diversity microbiota on colonization risk.

The construction of our mathematical model of *C. difficile* transmission would be impossible without data from laboratory experiments and population-based observational studies on antibiotic drug use, host microbiota diversity, and *C. difficile* colonization. Through consolidating this data, our model reveals that the effects of the microbiome state can create dramatic changes. The model predicts that reducing antibiotic drug use by <20% could halve endemic colonization. Such a reduction could be achieved by reducing unnecessary antibiotic drug use or shortening the duration of antibiotic drug courses when appropriate. Because antibiotic drug stewardship interventions have been shown to reduce antibiotic drug use in US long-term care facilities (*26*), reductions in the daily percentage of the nursing home resident population on antibiotic drugs could be feasible.

Incorporating the microbiome state might be necessary to accurately predict changes in pathogen colonization prevalence because of interventions such as antibiotic stewardship programs and pathogen reduction efforts (*27*). Accounting for those microbiota-mediated indirect effects, and reducing uncertainty in associated parameters, is increasingly necessary as new microbiome-targeted therapies emerge. That accounting is especially relevant when antibiotic drug use is unavoidable. Restoring microbiota diversity after necessary antibiotic drug treatment is a critical mechanism to leverage for reducing the susceptibility and infectivity that drives *C. difficile* transmission. Microbiome-based pathogen reduction interventions (e.g., fecal microbiota products and live biotherapeutic products) promote changes in microbial growth and work by reducing susceptibility (i.e., increasing colonization resistance) or selectively decolonizing body sites in the host to reduce infectivity and onward transmission (*25*). Our model provides a framework for evaluating the possible population-level effect of such interventions depending on which pathways they interrupt (e.g., duration of colonization, infectivity, microbiota diversity recovery), helping prioritize the treatments with the greatest potential to reduce colonization.

The first limitation of our study is that we did not distinguish between symptomatic *C. difficile* infection and asymptomatic colonization. Persons with symptomatic infection might have differences in infectivity, microbiota diversity recovery rate, duration of colonization, and antibiotic drug use compared with persons with asymptomatic colonization. Second, we have made assumptions about the relationships between low-diversity microbiota groups and increases in colonization susceptibility and infectivity. Although those mechanisms for increases are supported by literature (*6*,*20*,*21*), the chosen values are not directly supported by literature. We have chosen small ranges to avoid overstating the effect of antibiotic drugs on model outcomes. Third, we chose α diversity as our microbiota metric because of its ubiquity in the literature and generalizability across the nursing home population. Other measures of the microbiome might have higher predictive value (*28*) for colonization but require more individualized patient-level data. Fourth, we assumed a single linear relationship between antibiotic drug use over time in the nursing home population and disruption of the host microbiota. That relationship is likely nonlinear and dependent on drug dose, duration, or type (*29*–*33*). Previous work has linked specific antibiotic drugs to increased colonization risk (*23*,*34*), but accurately assessing the proportion of the population at an average nursing home receiving antibiotic drugs and the microbiome response remains challenging because of a lack of data. In addition, other medications might have adverse effects on the gut microbiota and affect *C. difficile* colonization risk (*35*). Fifth, we did not validate the modeled relationship between population-level antibiotic drug use and *C. difficile* colonization prevalence against empirical data (Figure 3). That limitation reflects a lack of paired data regarding antibiotic drug use and *C. difficile* colonization prevalence at different levels of antibiotic drug use. Our results should therefore be interpreted as model-based projections rather than empirically fitted results. Closing that gap is a key priority for future work; measuring prevalence of *C. difficile* colonization when antibiotic drug use is low could validate our findings (Figure 3) that small changes in antibiotic drug use can lead to large changes in colonization prevalence. Confirming that finding with empirical data would considerably strengthen the case for targeted stewardship interventions. Finally, we did not make distinction between competing *C. difficile* strains (e.g., nontoxigenic and toxigenic), which might yield different disease outcomes.

We built a mathematical model that elucidates the relationship between antibiotic drug use, gut microbiota diversity, and *C. difficile* colonization. To clarify the relationship between antibiotic drug use and colonization, we also quantified the relative effect of individual model parameters on *C. difficile* colonization burden. Two findings carry direct public health relevance. First, the relationship between population-level antibiotic drug use and colonization prevalence is nonlinear: our model predicts that, as antibiotic drug use approaches zero, there are larger effects on reduction in population-level colonization prevalence. Such a reduction suggests even small increases in stewardship initiatives could have large effects, especially in scenarios where antibiotic drugs use is already low. Second, because gut microbiota composition mediates that relationship, microbiota-targeting interventions that restore diversity offer a complementary mechanism for reducing colonization prevalence in situations where antibiotic drug use is unavoidable. Achieving those goals will require data collection of relevant epidemiologic data to validate our findings. Our results can help prioritize data collection, together with future modeling, to better characterize the cascading population-level effects of both microbiome-based pathogen reduction and antibiotic drug stewardship.

Appendix Additional information about reducing antibiotic drug use effects on *Clostridioides difficile* colonization in nursing home residents.
