# Supplementary material for: Reducing Antibiotic Drug Use Effects on Clostridioides difficile Colonization in Nursing Home Residents
Source: Emerg Infect Dis. 2026 Nov;32(11):1807–15. doi: 10.3201/eid3211.260743 (PMC13715871; doi:10.3201/eid3211.260743)
Supplement: Supplementary file 1 [file 26-0743-Techapp-s1.pdf]

Article DOI: https://doi.org/10.3201/eid3211.260743

EID cannot ensure accessibility for supplementary materials supplied by authors. Readers who have difficulty accessing supplementary content should contact the authors for assistance.

Reducing Antimicrobial Drug Use Effects on *Clostridioides difficile* Colonization in Nursing Home Residents

Appendix

Appendix Methods

Model equations

$$\frac{dU_H}{dt} = -\frac{\beta(C_H + \theta C_L)}{N}U_H - \alpha U_H + \delta U_L + \gamma C_H - \zeta U_H + \zeta N(1 - b_L)(1 - \mu)$$

$$\frac{dU_L}{dt} = -\frac{\beta(C_H + \theta C_L)}{N}\omega U_L + \alpha U_H - \delta U_L + \eta\gamma C_L - \zeta U_L + \zeta N(b_L)(1 - \mu)$$

$$\frac{dC_H}{dt} = \frac{\beta(C_H + \theta C_L)}{N}U_H - \alpha C_H + \phi\delta C_L - \gamma C_H - \zeta C_H + \zeta N(1 - b_L)(\mu)$$

$$\frac{dC_L}{dt} = \frac{\beta(C_H + \theta C_L)}{N}\omega U_L + \alpha C_H - \phi\delta C_L - \eta\gamma C_L - \zeta C_L + \zeta N(b_L)(\mu)$$

A visual representation of these equations in show in **Appendix Figure 1**.

Comparator Model

The comparator model is the same compartmental model described in **Appendix Figure 1** of the manuscript. However, instead of sampling from parameter range, a single value was chosen for each parameter set and transmission of C. difficile colonization was not affected by microbiota diversity (**Appendix Table**). Model parameters which were impacted by microbiota diversity (increased infectivity in low diversity microbiota group (θ), increase susceptibility in the low diversity microbiota group (ω), spontaneous decolonization in the low microbiota group (η), and the multiplier to microbiota diversity recover rate when colonized (ϕ)) were all set to one in this model parameterization. Similar to the model in the manuscript, β was calculated

such that endemic colonization was 10% when the daily percentage of the nursing home resident population on antimicrobial drugs was 8%.

Modeling fitting for equilibria dynamics

Parameter sets were uniformly sampled from literature-informed ranges (**Table in main text**) and the transmissibility parameter (β) was estimated. This procedure was repeated until there were 500 parameter sets. Once β was estimated, the daily percentage of the nursing home resident population on antimicrobial drugs was varied between 2% and 14% for each parameter set and the proportion colonized at equilibrium was calculated.

Modeling fitting for parameter effects and uncertainty

We used the same literature-informed parameter ranges to determine the change in equilibrium colonization prevalence and the reduction in the transmissibility parameter (β) required to offset an increase in any model parameter. The process described above was repeated for daily percentage of the nursing home resident population on antimicrobial drugs, modified to sample from a uniform distribution instead of held constant 8%. This procedure was repeated until there were 500 parameter sets. Then, within each of the 500 parameter sets, we individually increased a single parameter value by 5% compared to its baseline.

Modeling results

We analyzed the system at equilibrium, so all colonization prevalence estimates are equilibrium values. We used R statistical software with the deSolve and rootSolve packages to find steady state values of each compartment.

Calculating reduction in transmissibility shown in Figure 1C

We calculate the reduction in transmissibility by estimating β_{orig} for the original parameter set and then increasing each parameter by 5% one at a time and estimating β_{new} using the same endemic colonization used to estimate β_{orig}.

$$\text{transmissibility reduction goal} = \left(1 - \frac{\beta_{new}}{\beta_{orig}}\right) * 100$$

Defining "microbiota" and "the microbiome"

Our model seeks to understand effects of the microbiome, but a challenge is often we only have microbiota data. Thus, our terminology will vary depending on the scope. Specifically,

the microbiome encompasses all commensal, symbiotic, or pathogenic microorganisms (e.g., bacteria, fungi, viruses, and archaea), including their genetic and metabolic products, in a host or environment. Therefore, the "microbiome" is akin to the ecologic definition of an ecosystem in that it includes abiotic aspects as one would when defining a forest by specifying the type of soil and rainfall in addition to the plant species present. In contrast, "microbiota" describes only the ecologic community. Microbiota or community descriptions refer only to measures of species abundance. For pathogen interactions with the microbiome or microbiota, the key difference is that the microbiome might additionally quantify secondary metabolites, pH, degradation of the gut environment, or other measures of inflammation that could relate to susceptibility, infectivity, duration of colonization, or recovery of microbiota diversity through time.

Adapting the model to other pathogens of interest

We can use the same model structure (combined with different parameter values tailored to a new pathogen) to model other pathogens where we think host microbiota may have important effects on similar traits such as susceptibility to colonization.

Appendix Results

Comparator Model versus Main Model

The results from showing the comparator model versus the manuscript model are shown in **Appendix Figure 2**.

Sampled Model Parameter Space

The sampled model parameter space is shown in **Appendix Figure 3 and 4**.

References

1. Berkell M, Mysara M, Xavier BB, van Werkhoven CH, Monsieurs P, Lammens C, et al.; ANTICIPATE study group. Microbiota-based markers predictive of development of *Clostridioides difficile* infection. Nat Commun. 2021;12:2241. PubMed https://doi.org/10.1038/s41467-021-22302-0

2. van Werkhoven CH, Ducher A, Berkell M, Mysara M, Lammens C, Torre-Cisneros J, et al.; ANTICIPATE Study Group. Incidence and predictive biomarkers of *Clostridioides difficile* infection in hospitalized patients receiving broad-spectrum antibiotics. Nat Commun. 2021;12:2240. PubMed https://doi.org/10.1038/s41467-021-22269-y

3. Magill SS, O'Leary E, Ray SM, Kainer MA, Evans C, Bamberg WM, et al.; Emerging Infections Program Hospital Prevalence Survey Team. Assessment of the appropriateness of antimicrobial use in US hospitals. JAMA Netw Open. 2021;4:e212007. PubMed https://doi.org/10.1001/jamanetworkopen.2021.2007

4. Thompson ND, Stone ND, Brown CJ, Penna AR, Eure TR, Bamberg WM, et al. Antimicrobial use in a cohort of US nursing homes, 2017. JAMA. 2021;325:1286–95. PubMed https://doi.org/10.1001/jama.2021.2900

5. Anthony WE, Wang B, Sukhum KV, D'Souza AW, Hink T, Cass C, et al. Acute and persistent effects of commonly used antibiotics on the gut microbiome and resistome in healthy adults. Cell Rep. 2022;39:110649. PubMed https://doi.org/10.1016/j.celrep.2022.110649

6. Crobach MJT, Vernon JJ, Loo VG, Kong LY, Péchiné S, Wilcox MH, et al. Understanding *Clostridium difficile* Colonization. Clin Microbiol Rev. 2018;31:e00021–17. PubMed https://doi.org/10.1128/CMR.00021-17

7. Donskey CJ, Kundrapu S, Deshpande A. Colonization versus carriage of *Clostridium difficile*. Infect Dis Clin North Am. 2015;29:13–28. PubMed https://doi.org/10.1016/j.idc.2014.11.001

8. Wolford H, Khan MA, Paul P, Toth DJA, Octaria R, Healy JM, et al. Projected impact of a pathogen reduction intervention on carbapenem-resistant Enterobacterales bloodstream infections and deaths in the United States. Infect Control Hosp Epidemiol. 2026;47:476–82. PubMed https://doi.org/10.1017/ice.2020.1236

Appendix Table. Full parameter table for comparator model of *C. difficile* colonization transmission in nursing home residents.

Description	Variable/ parameter notation	Comparison value (units)	Source
Initial proportion of population and newly admitted proportion of population with low-diversity microbiota	b_L	0.25	(*1*,*2*)
Total population size	N	100	Assumed
Proportion of newly admitted nursing home residents that are colonized	μ	0.01	Assumed
Transmission rate	β	–	Fitted value
Relative increase in infectivity due to a lower diversity microbiota (i.e., infectivity of $C_L > C_H$)	θ	1	Assumed
Relative increase in susceptibility to colonization for low-diversity microbiota group	ω	1	Assumed
Indicator for whether the low diversity microbiota group can spontaneously decolonize	η	1	Assumed
Antibiotic-induced transition rate from high to low-diversity microbiota, 1/days	$\alpha*$	0.013	Estimated

Description	Variable/ parameter notation	Comparison value (units)	Source
Daily nursing home resident population on antimicrobial drugs, %	abx	8%	(*3*,*4*)
Days to go from high to low diversity upon starting antibiotics, days	ψ	6	(*5*)
Microbiota diversity recovery rate from low diversity to high diversity, for the uncolonized group, 1/days	δ †	0.048	Estimated
Daily nursing home resident population not on antimicrobial drugs, %	$1 - abx$	92%	(*3*,*4*)
Days to recover from low to high diversity upon discontinuing antibiotics, days	r_d	19	(*5*)
Duration of *C. difficile* colonization, days	$\frac{1}{\gamma}$	80	(*6*,*7*)
Multiplier to microbiota diversity recovery for the colonized group	ϕ	1	Assumed
Average length of nursing home stay, days	$\frac{1}{\zeta}$	165	(*8*)

⁎α = (daily proportion of the NH resident population on antibiotics) x (1/ days to go from high to low diversity upon starting antibiotics)
†δ = (daily proportion of the NH resident population not on antibiotics) x (1/ days to recover from low to high diversity upon discontinuing antibiotics)

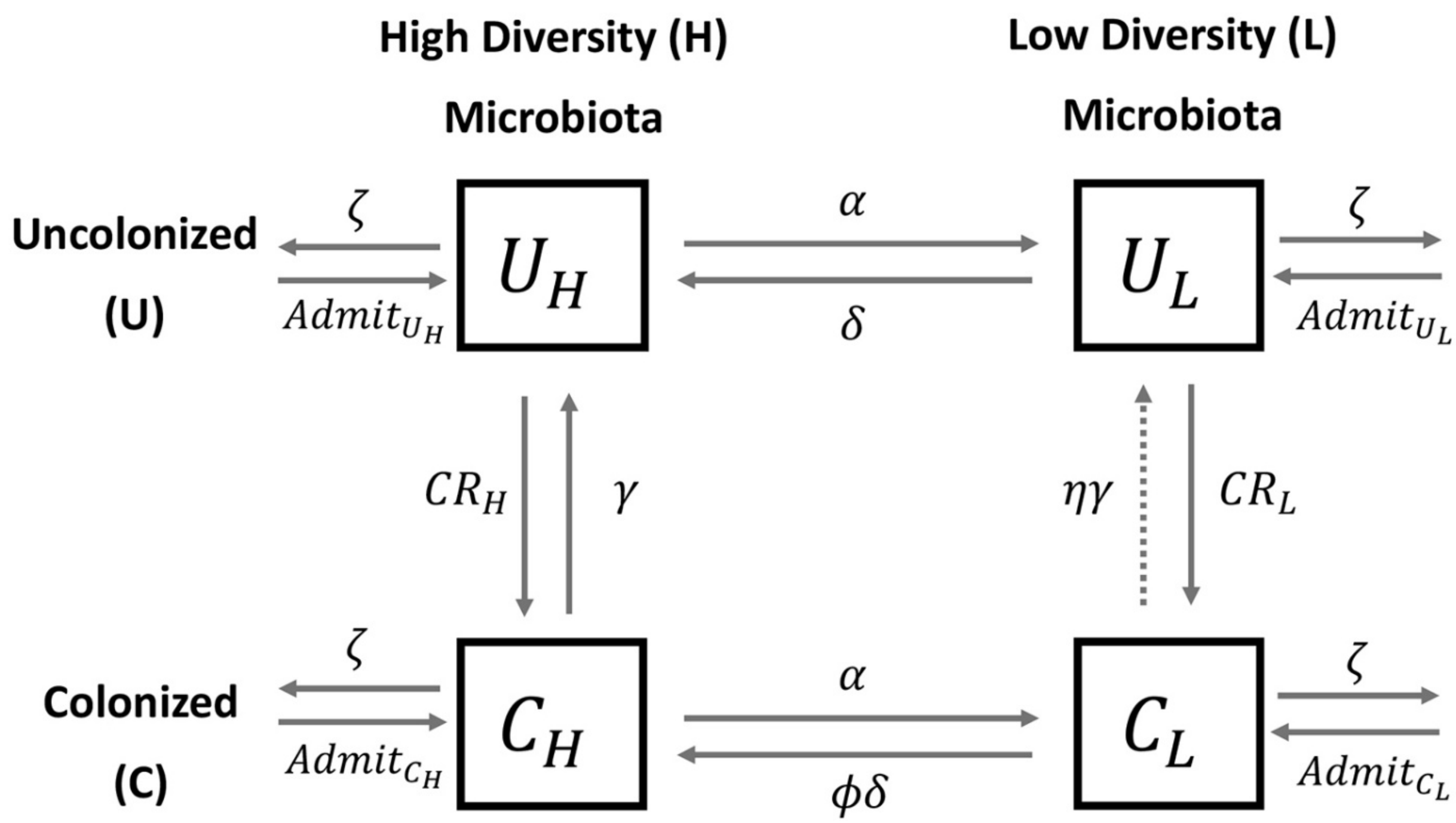

Appendix Figure 1. Flow diagram of compartmental model in study of indirect effects of antibiotic use on the microbiome state and the implications for endemic *C. difficile* colonization and transmission in nursing homes. Arrows denote transitions between population states of uncolonized (U) and colonized (C) groups with high diversity (H subscript) and low diversity (L subscript) microbiota α diversity. “CR” = colonization rate which is a function of transmission rate (β) with possibility for increased susceptibility (ω) and

infectivity (θ) based on microbiota diversity. Transitions from high to low diversity microbiota groups (α) are proportional to the daily percentage of the NH resident population on antibiotics. The recovery from the low microbiota diversity is controlled by the microbiota diversity recovery rate (δ). The recovery rate can decrease in the colonized group (ϕ). The colonized high diversity microbiota group can spontaneously decolonize (γ), but not the colonized low diversity microbiota group ($\eta = 0$). Spontaneous decolonization of the low diversity microbiota group is possible in the comparator model ($\eta = 1$) described in **Appendix Methods**. The discharge rate (ζ), calculated by inverting the average length of stay, from each group is the same. Admissions into the different groups are calculated from the base proportion of population with low diversity microbiota (b_L), the proportion of newly admitted nursing home residents that are colonized (μ), and the discharge rate (ζ).

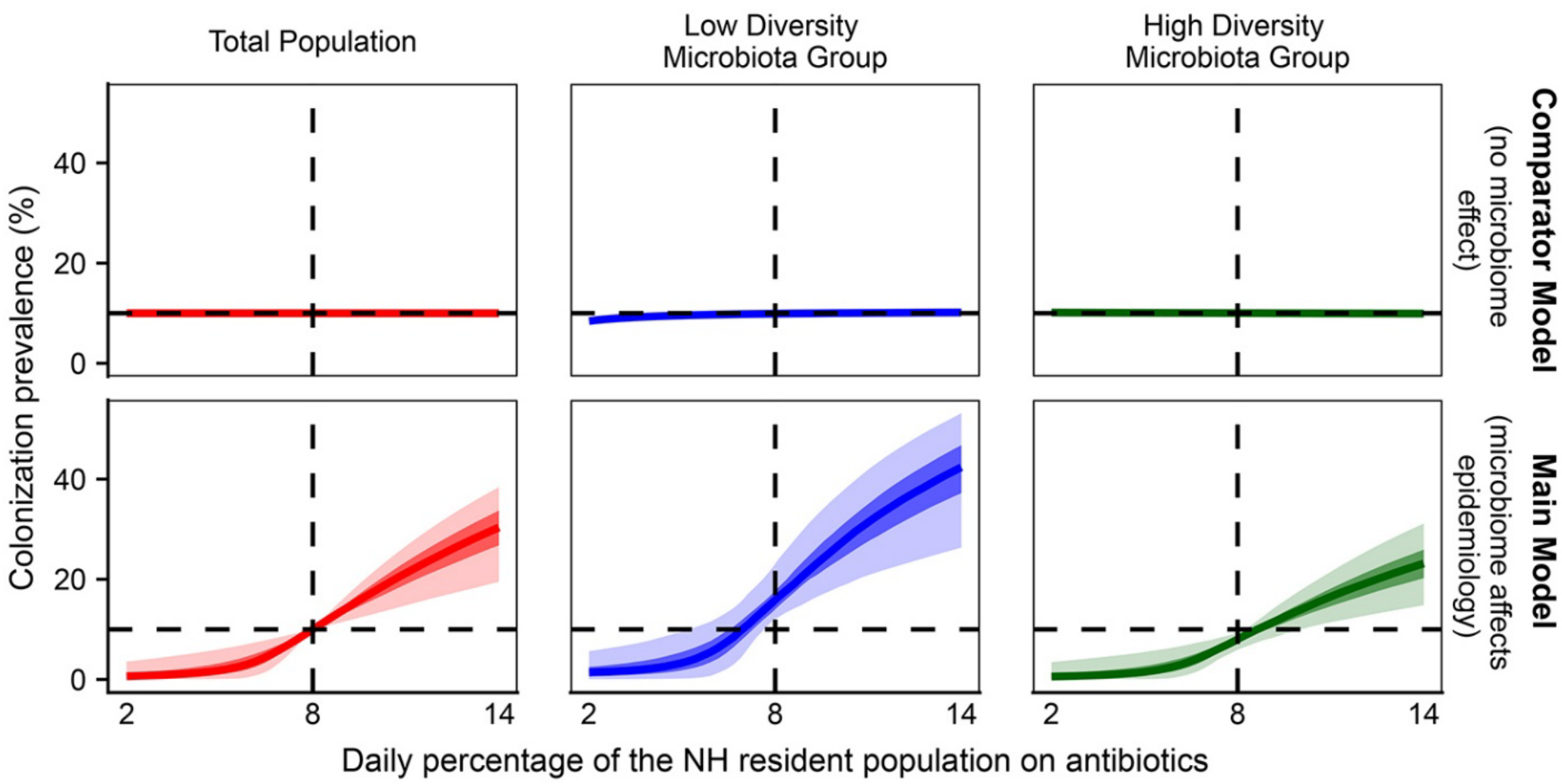

Appendix Figure 2. Model results of comparator model and main model from study of the indirect effects of antibiotic use on the microbiome state and the implications for endemic *C. difficile* colonization and transmission in nursing homes. Endemic *C. difficile* colonization assuming 10% total colonization prevalence (dashed horizontal line) when daily population-level antibiotic use is 8% (dashed vertical line). The darker and lighter shaded regions, respectively, show the central 50% and 95% simulation intervals, and the darkest solid lines depict median values. Results for the total population are shown in red, results for the low microbiota diversity group are shown in blue, and results for the high microbiota diversity group are shown in green.

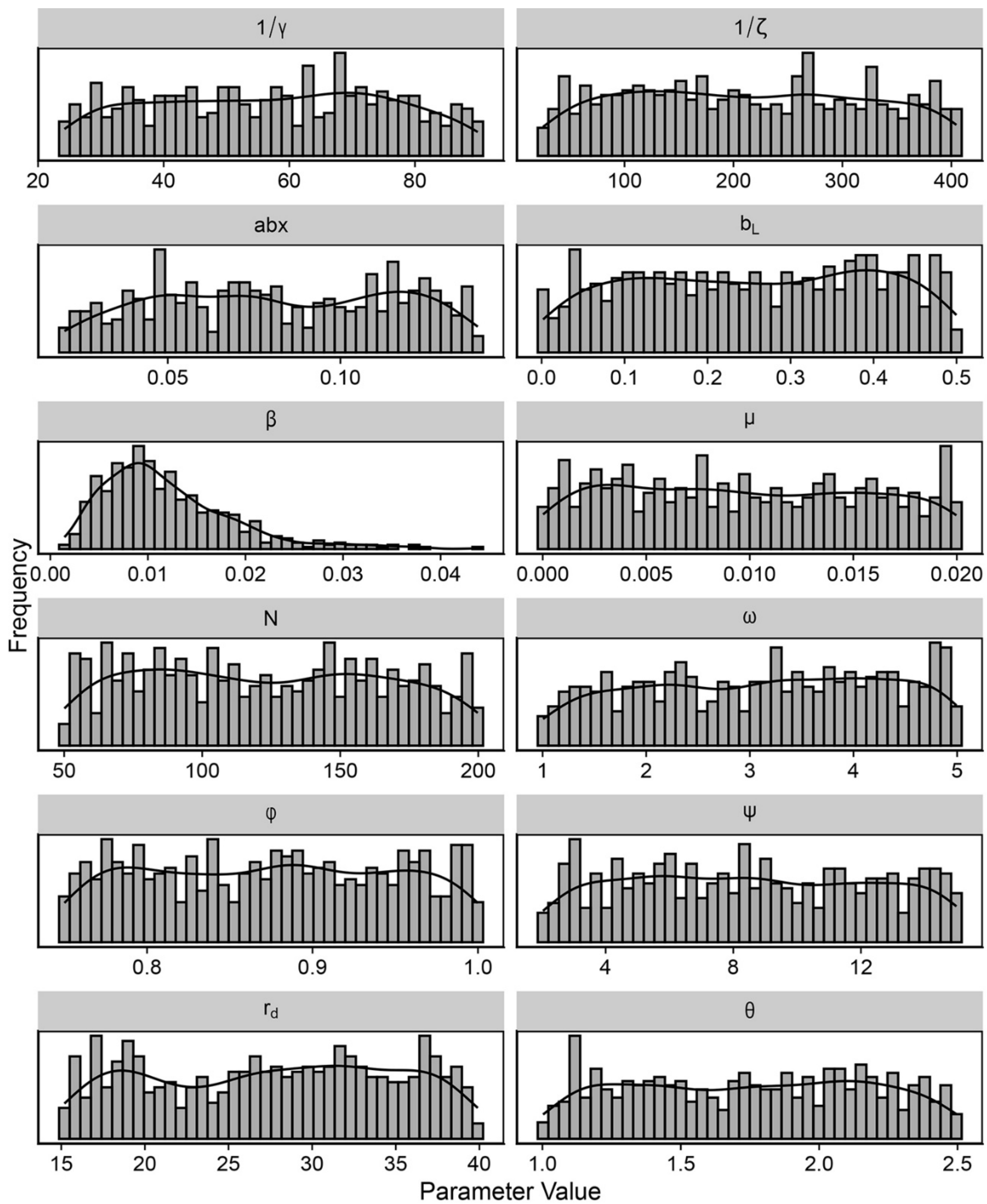

Appendix Figure 3. Model parameters were sampled from uniform distributions in study of indirect effects of antibiotic use on the microbiome state and the implications for endemic *C. difficile* colonization and transmission. The resulting parameter set roughly follows uniform distributions. Note: The microbiota recovery rate (δ) was not sampled, the days to recover from low to high microbiota diversity upon discontinuing antibiotics and daily proportion of the nursing home resident population on antimicrobial drugs were sampled and the microbiota recovery rate was calculated using the two parameters. $1/\gamma$ –

Duration of C. difficile colonization, $1/\zeta$ – average length of nursing home stay, abx – daily proportion of the nursing homeresident population on antimicrobial drugs, b_L- Initial proportion of population and newly admitted proportion of population with low diversity microbiota, β – transmission rate, μ – Proportion of newly admitted nursing home residents that are colonized, N – total population size, ω – relative increase in susceptibility to colonization for low-diversity microbiota group, ϕ – multiplier to microbiota diversity recovery for the colonized group, ψ – Days to go from high to low diversity upon starting antibiotics, r_d – Days to recover from low to high diversity upon discontinuing antibiotics, θ – Relative increase in infectivity due to a lower diversity microbiota

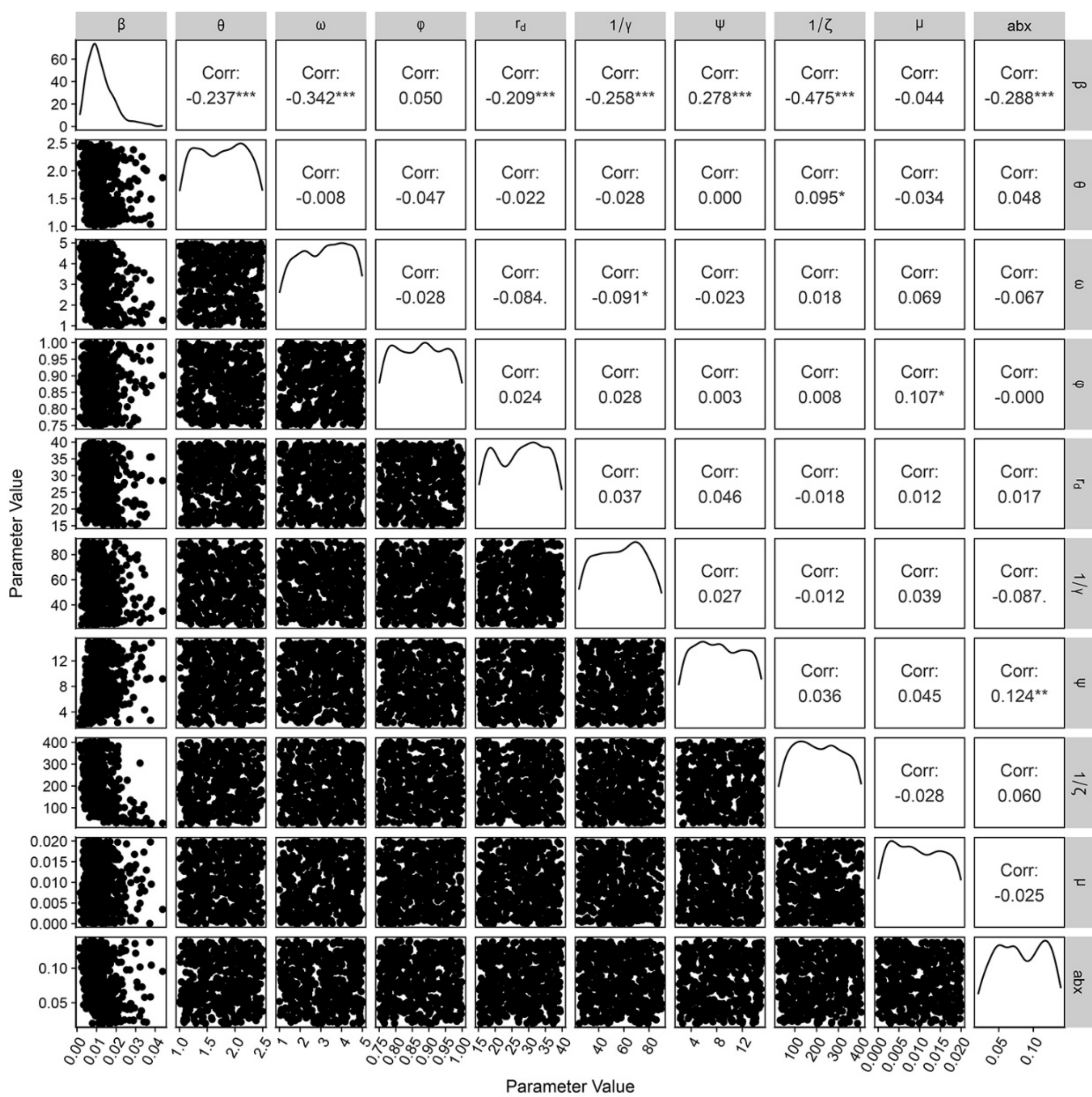

Appendix Figure 4. Correlation plots of some model parameters in study of indirect effects of antibiotic use on the microbiome state and the implications for endemic *C. difficile* colonization and transmission. The transmissibility parameter (β) is weakly negatively correlated with susceptibility (ω), days to

decolonization ($1/\gamma$), length of stay ($1/\zeta$), and the daily proportion of the nursing home resident population on antimicrobial drugs (abx). The transmissibility parameter (β) is weakly positively correlated with days to microbiota dysbiosis (ψ). β – transmission rate, θ – Relative increase in infectivity due to a lower diversity microbiota, ω – relative increase in susceptibility to colonization for low-diversity microbiota group, ϕ – multiplier to microbiota diversity recovery for the colonized group, r_d – Days to recover from low to high diversity upon discontinuing antibiotics, $1/\gamma$ – Duration of C. difficile colonization, ψ – Days to go from high to low diversity upon starting antibiotics, $1/\zeta$ – average length of nursing home stay, μ – Proportion of newly admitted nursing home residents that are colonized, abx – daily proportion of the NH resident population on antibiotics
